# Veterinary method evaluation of Vitek-2 compact for antimicrobial susceptibility testing of *Staphylococcus* spp. and *Enterococcus* spp.

**DOI:** 10.1128/jcm.00961-25

**Published:** 2025-12-17

**Authors:** Sarah Gefroh, Briena Meier, Kelli Maddock

**Affiliations:** 1 Veterinary Diagnostic Laboratory, North Dakota State University 3323, Fargo, North Dakota, USA; University of California, Davis, California, USA

**Keywords:** *Staphylococcus pseudintermedius*, one health, clinical veterinary microbiology, antimicrobial susceptibility testing

## Abstract

Quality laboratory data are central to antimicrobial resistance detection in support of good patient care and for use in a One Health surveillance system. Here, we evaluated the Vitek-2 Compact AST-GP81 cards against Sensititre COMGP1F broth microdilution panels. A total of 51 *Staphylococcus* spp. not *aureus/lugdunensis;* 30 *Staphylococcus aureus* and *Staphylococcus lugdunensis*; and 34 *Enterococcus* spp. were selected for testing. Overall performance of the Vitek-2 Compact was acceptable, with at least 96% essential agreement and 94% categorical agreement across organism groups, exceeding the minimum performance goal of ≥90% agreement; however, several antimicrobials did not meet minimum performance standards. For *Staphylococcus* spp. not *aureus/lugdunsis,* minocycline failed entirely, precluding patient reporting and clindamycin required offline confirmatory testing. We observed unacceptable rates of minor errors if *Staphylococcus* cephalosporin breakpoints were used, whereas a surrogate agent produced consistent results between methods. Notably, we reaffirm that it is necessary to confirm any susceptible *Staphylococcus pseudintermedius* penicillin results with an induced nitrocefin β-lactamase test, especially if Sensititre COMPGP1F panels are used. Both test panels require updated antimicrobial offerings and dilution ranges to ensure a full range of test results can be reported. These updates should include consideration for test ranges that allow for on-scale quality control testing and translation across One Health sectors. Because antimicrobial susceptibility test results impact far more than single patient care in veterinary medicine, including human, herd, and environmental health, we advocate for the use of stringent antimicrobial susceptibility test method evaluation procedures in veterinary laboratories.

**IMPORTANCE:**

Antimicrobial resistance is a critical threat to human and animal health globally. While our patient populations are different, we are connected by our shared environments and intertwined existence. As such, our antimicrobial susceptibility testing diagnostic and surveillance tools, as well as methods to evaluate their performance, should be uniform and capable of detecting critical antimicrobial resistance mechanisms. We evaluated the Vitek-2 Compact against our legacy Sensititre system and determined that accurate patient results and quality surveillance data could be produced using the Vitek-2 Compact. We used the evaluation method described in the Clinical Microbiology Procedures Handbook to demonstrate the utility of this method to peer veterinary laboratories. This publication importantly demonstrates that this evaluation procedure is scalable for veterinary applications. Furthermore, there is a need for updates to veterinary test panels as well as investments in and advancement of veterinary diagnostic tools, in support of a One Health approach to antimicrobial resistance detection.

## INTRODUCTION

Antimicrobial susceptibility testing (AST) is a critical treatment and surveillance tool for detection of antimicrobial resistance (AMR) in clinically significant bacteria isolated from human and veterinary patients. One Health is “an integrated, unifying approach that aims to sustainably balance and optimize the health of people, animals, and ecosystems.” (1) Collaboration between each of these sectors is necessary to address issues of mutual concern, such as infectious disease and AMR. Veterinary laboratories play a significant role in detecting (2–5) zoonotic pathogens and tracking AMR that can be translated across the One Health spectrum. To provide the most useful data, it is necessary to ensure that our diagnostic tools perform and are evaluated similarly across sectors and that organisms and clinically significant resistance mechanisms can be detected equally. However, development and support of veterinary diagnostic tools have been assigned a lower priority. As such, veterinary AST data may not be equivalent to those generated in human diagnostics.

Accredited (6) veterinary laboratories are familiar with test method evaluation requirements for laboratory tests; however, from discussions with peer laboratories, we believe many are unfamiliar with resources widely used in human laboratories for the evaluation of AST devices, such as those published in the Clinical Microbiology Procedures Handbook (7) or Clinical and Laboratory Standards Institute (CLSI) Guideline M52: Verification of Commercial Microbial Identification and Antimicrobial Susceptibility Testing Systems (8). These resources are important because they include additional considerations for evaluation of AST methods not addressed by standard test validation criteria in veterinary medicine. This includes essential and categorical agreement of minimal inhibitory concentration (MIC) results between test methods, quality control (QC) performance, and categorization of errors based on the potential clinical impact those errors might have.

Deprioritization of veterinary diagnostic tools and financial constraints of veterinary laboratories have led to few commercially available veterinary AST products for laboratories to choose from and less urgency to provide panel updates. As such, antimicrobial dilutions on a test panel may be obsolete or inadequate (9), barring evaluation of updated clinical breakpoint performance and making method comparisons difficult. In some instances, on-scale QC may not be attainable (9) or the manufacturer-recommended QC procedures differ from those used for patient isolates (10), making it difficult to troubleshoot or critically evaluate test performance. Additionally, veterinary laboratories may lack adequate resources for extensive verification of a test device due to cost or lack of access to reference strains of veterinary pathogens and antimicrobials. Together, these challenges may further stall laboratory uptake of new clinical breakpoints, directly impacting patient care and AMR detection.

Application and interpretation of data obtained by veterinary AST panels may further complicate AMR detection across the One Health spectrum. Clinical breakpoints differ between humans and different animal species. When only veterinary-specific breakpoints are included on commercial AST panels, it can be difficult to detect critical resistance mechanisms or accurately assess resistance rates observed among clinical isolates from human and veterinary patients. In addition, some CLSI veterinary breakpoints are intended to call most or all isolates of a bacterial species resistant to a particular antimicrobial, e.g., staphylococci and chloramphenicol (11), to signal to a veterinarian not to use that treatment in an animal species (12, 13). In some instances, veterinary-specific breakpoints exist where other sectors may use surrogate agents, inhibiting equivalent comparisons. These factors make surveillance data difficult to translate and may lead to misunderstandings surrounding rates of resistance between species, and such breakpoints make method evaluation even more challenging or impossible to accomplish.

The purpose of this study was to evaluate Vitek 2 Compact AST-GP81 card performance against Sensititre COMPGP1F dried broth microdilution (BMD) panels for *Staphylococcus* species (*Staphylococcus pseudintermedius, Staphylococcus felis, Staphylococcus epidermidis); Staphylococcus aureus* and *Staphylococcus lugdunensis;* and *Enterococcus* species. This study uniquely demonstrates the applicability of human-based guidelines in veterinary AST evaluation and highlights significant challenges associated with current clinical breakpoints, resource availability, diagnostic tools, and translatability across the One Health spectrum.

## MATERIALS AND METHODS

### Bacterial isolates

A total of 51 *Staphylococcus* spp. not *aureus/lugdunensis;* 30 *Staphylococcus aureus* and *Staphylococcus lugdunensis*; and 34 *Enterococcus* spp. were selected for testing. Efforts were made to ensure isolates would represent a variety of resistance patterns, but unusual resistance mechanisms were difficult to account for because well-characterized veterinary reference panels are currently unavailable to accommodate all resistance patterns. Isolates were recovered from clinical and surveillance specimens submitted to the NDSU Veterinary Diagnostic Laboratory from August 2020 through April 2025; fresh or frozen isolates were used for this study. Frozen isolates were stored in 15% tryptic soy broth and glycerol (Hardy Diagnostics, Santa Maria, CA). Fresh isolates were subcultured once for purity and frozen isolates were subcultured twice to 5% sheep blood agar (BAP, Hardy Diagnostics, Santa Maria, CA) and incubated at 35°C in 5% CO_2_ for 18–24 h prior to testing.

### Antimicrobial susceptibility testing 

Each isolate was tested simultaneously with both methods using well-isolated colonies from one BAP. The Vitek-2 Compact and Sensititre methods require different media, 0.45% saline and demineralized water, respectively, for the initial 0.5 McFarland preparation, so we were unable to use the same inoculum for both methods. Purity plates were prepared from the positive control well of the BMD panel and the inoculum tube for the Vitek panel and incubated for 18–24 h prior to assessment for pure growth.

### Sensititre COMPGP1F

Sensititre BMD COMPGP1F panels, our reference method, were performed on canine and feline patient isolates according to manufacturer’s instructions for use (IFU) (10). QC for *S. aureus* ATCC 29213 and *Enterococcus faecalis* ATCC 29212 was performed in the same manner as patient isolates (30 µL transfer volume), which is considered best practice to thoroughly evaluate test methodology (14), technologist performance, and reduce opportunities for error (15). This deviates from the manufacturer’s IFU (10) (10 µL transfer volume) but was assessed in-house in a previous study, resulting in acceptable QC results and appropriate colony counts. The panels were automatically read using BIOMIC V3, software version 7.9.2.2023, with results manually confirmed by microbiology staff.

### Vitek 2 compact

QC and patient isolates for Vitek 2 Compact were tested using AST-GP81 cards according to the manufacturer’s IFU (16), software version 9.04, and CLSI VET01S 7th edition (11).

### Interpretive criteria

Canine and feline specific breakpoints were used when available, otherwise, human breakpoints were used as appropriate (Table 1). Veterinary breakpoints were derived from CLSI VET01S 7th edition (11) and human breakpoints from CLSI M100 35th edition (17). While our laboratory routinely uses surrogate agent testing (oxacillin, cefoxitin) for staphylococci, we also chose to evaluate agreement of amoxicillin-clavulanate and cephalosporin (cefalothin, cefpodoxime, and cefovecin) MICs between the methods in addition to performance of oxacillin; however, results of these antimicrobials were not included in final performance calculations.

**TABLE 1:** Breakpoints evaluated in this study^*a*^

Antimicrobial	S	I	SDD	R	Breakpoint used	Animal host
	MIC (µg/mL)					
All Staphylococci						
Penicillin	≤0.12			≥0.25	H	C, F
Amoxicillin/clavulanic acid	≤0.25/0.12	0.5/0.25		≥1.0/0.5	C, F	C, F
Enrofloxacin	≤0.06		0.12–0.25	≥0.5	C	C
	≤0.5	1–2		≥4	F	F
Minocycline	≤0.5	1		≥2	C	C, F
Marbofloxacin	≤0.12		0.25	≥0.5	C	C
	≤1	2		≥4	F	F
Amikacin	≤4	8		≥16	C	C, F
Gentamicin	≤4	8		≥16	H	C, F
Erythromycin	≤0.5	1–4		≥8	H	C, F
Clindamycin	≤0.5	1–2		≥4	C	C, F
Doxycycline	≤0.12	0.25		≥0.5	C	C, F
Chloramphenicol	≤2	4		≥8	C	C, F
Trimethoprim/sulfamethoxazole	≤2/38			≥4/76	H	C, F
*Staphylococcus pseudintermedius* and *S.aureus*						
Cefalothin	≤2	4		≥8	C	C
Cefpodoxime	≤2	4		≥8	C	C

*Staphylococcus pseudintermedius, aureus* and *felis*						
Pradofloxacin	≤0.25	0.5–1		≥2	F	F
*Staphylococcus pseudintermedius* only						
Cefovecin	≤0.5	1		≥2	C	C, F
Pradofloxacin	≤0.25	0.5–1		≥2	C	C
*Staphylococcus pseudintermedius/schleiferi/epidermidis/felis*						
Oxacillin	≤0.25			≥0.5	VET01S 7th ed^*b*^	C, F
*Staphylococcus aureus and S. lugdunensis*						
Oxacillin	≤2			≥4	H	C, F
*Staphylococcus* spp. not *aureus, lugdunensis, epidermidis, pseudintermedius, schleiferi*						
Oxacillin	≤0.5			≥1	H	C, F
*Enterococcus* spp.						
Penicillin	≤8			≥16	H	C, F
Erythromycin	≤0.5	1–4		≥8	H	C, F
Doxycycline	≤4	8		≥16	H	C, F
Nitrofurantoin	≤32	64		≥128	H	C, F
Chloramphenicol	≤2	4		≥8	C	C, F

^*a*^ C = Canine; F = Feline; H = Human; I = Intermediate; S = Susceptible; SDD = Susceptible, dose dependent; R = Resistant.

^*b*^ Not species-specific. Differs from CLSI M100 breakpoint for *S. pseudintermedius.*

### Panel limitations

Due to limitations of one or both methods’ calling ranges, some antimicrobials could not be fully evaluated (Table 2) and were, therefore, not validated for patient use.

**TABLE 2:** Summary of Sensititre COMPGP1F and Vitek 2 Compact AST-GP81 panel interpretation and/or calling range limitations^*a*^

Antimicrobial	Interpretive criteria^*b*^	Sensititre COMPGP1F calling range (µg/mL)	Vitek 2 Compact calling range (µg/mL)	Limitations
Staphylococci				
Amikacin	≤4 S	16–32	2–64	Sensititre unable to detect S and I
	8 I			
	≥16 R			
Amoxicillin-Clavulanic acid-Canine and feline	≤0.25/0.12 S	0.25–8	2–32	Vitek unable to detect S and I
	0.5/0.25 I			
	≥1.0/0.5 R			
Doxycycline-Canine	≤0.12 S	0.12–0.5	0.5–16	Vitek unable to detect S and I
	0.25 I			
	≥0.5 R			
Enrofloxacin-Canine	≤0.06 S	0.25–4	0.5–4	Sensititre unable to detect S; Vitek unable to detect S or SDD
	0.12–0.25 SDD			
	≥0.5 R			
Marbofloxacin-Canine	≤0.12 S	1–4	0.5–4	Sensititre and Vitek unable to detect S and SDD
	0.25 SDD			
	≥0.5 R			
Enterococci and Staphylococci				
Chloramphenicol-Canine	≤2 S	8–32	4–64	Sensititre unable to detect S and I; Vitek unable to detect S
	4 I			
	≥8 R			

^*a*^ I = Intermediate; R = Resistant; S = Susceptible; SDD = Susceptible, Dose Dependent.

^*b*^ All breakpoints are from CLSI VET01S, 7th edition.

### Induced nitrocefin test for detection of β-lactamase production

*Staphylococcus* spp. susceptible to penicillin (MIC ≤ 0.12 µg/mL) (17) by either AST method were confirmed by testing for β-lactamase production in accordance with VET01S guidelines (11), using a Hardy Nitrocef disk to manufacturer’s instructions (Hardy Diagnostics, Santa Maria, CA) and by the CLSI induced nitrocefin-based test method (17).

### Kirby Bauer and inducible clindamycin resistance testing

 *Staphylococcus* spp. susceptible or intermediate to clindamycin (MIC < 2 µg/mL) and resistant to erythromycin (>8 µg/mL) by the Sensititre system were also tested by D-test for inducible clindamycin resistance following the CLSI method described in M100 (17). Additionally, canine*-*specific clindamycin interpretations were determined by Kirby Bauer method following manufacturer instructions (Hardy Diagnostics, Santa Maria, CA) and CLSI VET01S interpretive criteria (11).

### Data analysis and resolution of discrepancies

Performance verification of the Vitek 2 Compact was conducted as described in the Clinical Microbiology Procedures Handbook (7). To demonstrate proficiency, reduce QC from daily to weekly, and to satisfy precision and reproducibility requirements, the Vitek 2 Compact was initially verified by performing the 3- by-5 replicate protocol (7, 8) where each QC strain is tested in triplicate for five consecutive days. While human clinical laboratories are accredited by the College of American Pathologists (CAP) or regulated by the Clinical and Laboratory Improvement Act (CLIA) guidelines, veterinary laboratories are not. Therefore, a full individual quality control plan (IQCP) was not required for reducing the frequency of QC testing.

Accuracy was assessed by an internal, side-by-side laboratory comparison of both methods. Following testing, essential agreement (EA) was evaluated by comparing numerical MIC values (acceptable within ± 1 dilution between methods); and categorical agreement (CA) was evaluated by comparing the interpretation (susceptible, intermediate, susceptible-dose dependent, and resistant). EA and CA errors were calculated as previously described (7) but are summarized as follows: Very major errors (VME) were calculated by dividing the number of very major errors by the total number of resistant or non-susceptible isolates while major errors (ME) were determined by dividing the number of major errors by the total number of susceptible isolates. Minor errors (mE) were derived from the total number of minor errors divided by the total number of isolates tested. To assess for random user error and as recommended for method evaluations (7), isolates with VMEs and MEs were repeated by both methods in duplicate. If the error was resolved after repeat testing, the initial result was discarded and not considered an error. Tertiary tests including induced and uninduced nitrocefin, inducible clindamycin resistance tests and Kirby-Bauer disks, were also used as discrepancy resolution methods. If the error was not resolved, it was included in the final error calculations. Antimicrobials with CA and/or EA of > 90% were considered to have achieved acceptable accuracy, and results from these antimicrobials were deemed appropriate for patient reporting. Antimicrobials with ≥3% VME and/or ME were considered invalid and are not included in patient reports. Acceptance criteria for mEs were ≤10%.

## RESULTS

### Vitek quality control

For 3 × 5 replicate testing, QC strains achieved acceptable results within the requisite +1 doubling dilution at least 95% of the time, and no results were out of CLSI-approved control ranges. Acceptable performance indicated that we could move from daily to weekly QC testing.

### Summarized test performance

 After error resolution, overall test performance was acceptable across organism groups (Table 3).

**TABLE 3:** Overview of test performance for canine and feline breakpoints after resolution of testing discrepancies^*a*^^,^^*b*^

Organism group	Total canine and feline isolates with MICs	Total canine and feline interpretations	Total S results	Total I results	Total R results	EA % (*n*/*N*)	CA % (*n*/*N*)	VME % (*n*/*N*)	ME % (*n*/*N*)	mE % (*n*/*N*)
*Staphylococcus* spp. (not *aureus/lugdunensi*s)	1,036	800	495	15	290	97.8 (1,013/1,036)	98.1 (785/800)	0.33 (1/305)	0 (0/495)	1.75 (14/800)
*Staphylococcus aureus/lugdunensis*	538	422	389	0	33	99.6 (536/538)	98.6 (416/422)	0 (0/33)	0.26 (1/389)	1.18 (5/422)
*Enterococcus* spp.	162	117	87	25	5	96.3 (156/162)	94.9 (111/117)	0 (0/30)	0 (0/87)	5.1 (6/117)

^*a*^ Individual test performance for *Staphylococcus* spp. cephalosporin and amoxicillin-clavulanate results and 3 *Enterococcus* spp. doxycycline isolate results were excluded from this summary.

^*b*^ CA = Categorical agreement; EA = Essential agreement; I = Intermediate; ME = Major error; mE = Minor error; MIC = Minimal inhibitory concentration; S = Susceptible; R = Resistant; VME = Very major error.

### *Staphylococcus* spp. (not *S. aureus* or *S. lugdunensis*) group

Fifty-one isolates were tested in the *Staphylococcus* spp. not *S. aureus/lugdunensis* group (Table 4), *S. pseudintermedius* (*n* = 45)*, S. felis* (*n* = 3)*, S. schleiferi* (*n* = 2), and *S. epidermidis* (*n* = 1). Of the initial 26 mEs, 12 were due to discrepancies in cephalosporin (cefovecin, cefpodoxime, and cefalothin) interpretations between methods. Conversely, using only surrogate agent (oxacillin) inference rules, the overall CA was 100%, suggesting that individual cephalosporin performance is inferior to oxacillin. Additionally, five isolates had penicillin MICs of ≤0.06 µg/mL (susceptible) by Sensititre COMPGP1F and tested 0.25 µg/mL or greater (resistant) by Vitek 2 Compact, resulting in 5 MEs. Additional testing for β-lactamase production was used for discrepancy resolution (Table 5).

**TABLE 4:** *Staphylococcus* spp. (not *S. aureus* or *S. lugdunensis*) group results^*a*^^,*b*,*f*^

Antimicrobial	Total	No. of isolates					Number (%)				
		S	I	SDD	R	NI	EA	CA	VME	ME	mE
Penicillin^*c*^-initial	51	12	0	0	39	0	96.1	100	0 (0)	**5** (**41.7**)	0 (0)
Penicillin^*c*^-resolved	51	12	0	0	39	0	96.1	90.2	0 (0)	0 (0)	0 (0)
Amoxicillin/Clavulanate	51	0	0	0	7	44	98	100	0 (0)	NA	0 (0)
Oxacillin^*c*^	51	34	0	0	17	0	90.2	100	0 (0)	0 (0)	0 (0)
Cefalothin^*e*^	45	40	0	0	5	0	97.8	97.8	0 (0)	0 (0)	1 (2.2)
Cefpodoxime^*e*^	45	36	1	0	8	0	91.1	88.9	0 (0)	0 (0)	**5** (**11.1**)
Cefovecin^*e*^	45	31	6	0	8	0	91.1	86.7	0 (0)	0 (0)	**6** (**13.3**)
Amikacin	51	0	0	0	0	51	100	NA	0 (0)	0 (0)	0 (0)
Gentamicin^*d*^	51	39	4	0	8	0	100	96.1	0 (0)	0 (0)	2 (3.9)
Enrofloxacin-Canine	51	0	0	0	15	36	100	100	0 (0)	0 (0)	0 (0)
Enrofloxacin-Feline	51	36	1	0	14	0	100	96.1	0 (0)	0 (0)	2 (3.9)
Marbofloxacin-Canine	51	0	0	0	13	38	100	100	0 (0)	0 (0)	0 (0)
Marbofloxacin-Feline	51	37	0	0	14	0	100	98	0 (0)	0 (0)	1 (1.9)
Pradofloxacin-Canine	51	37	3	0	11	0	100	98	0 (0)	0 (0)	1 (1.9)
Pradofloxacin-Feline	51	38	4	0	9	0	100	98	0 (0)	0 (0)	1 (1.9)
Erythromycin^*d*^	51	31	0	0	20	0	100	100	0 (0)	0 (0)	0 (0)
Clindamycin	50	34	1	0	15	0	92	94	1 (**6.6**)	0 (0)	2 (3.9)
Doxycycline	51	0	0	0	24	27	100	100	0 (0)	NA	0 (0)
Nitrofurantoin^*d*^	51	51	0	0	0	0	100	100	NA	0 (0)	0 (0)
Chloramphenicol	51	0	0	0	11	40	98	100	0 (0)	NA	0 (0)
Trimethoprim/Sulfamethoxazole^*d*^	51	37	0	0	14	0	100	100	0 (0)	0 (0)	0 (0)
Minocycline	35	19	2	0	14	0	97.1	85.7	0 (0)	0 (0)	**3** (**14.3**)

^*a*^ Penicillin discrepancies were resolved using induced and uninduced nitrocefin testing. Clindamycin and minocycline errors did not resolve with repeat testing.

^*b*^ CA = Categorical agreement; EA = Essential agreement; I = Intermediate; ME = Major error; mE = Minor error; MIC = Minimal inhibitory concentration; NA = Not applicable; S = Susceptible; SDD = Susceptible, dose dependent; R = Resistant; VME = Very major error. Results based on canine breakpoints unless otherwise specified.

^*c*^ Surrogate agent.

^*d*^ Human breakpoints.

^*e*^ *Staphylococcus pseudintermedius* only.

^*f*^ Boldface values indicate errors above acceptance limits.

**TABLE 5:** Discrepancy resolution for Canine *Staphylococcus pseudintermedius* penicillin and β-lactamase results^*a*^

Sample	Sensititre MIC (µg/mL)	Sensititre interpretation	Vitek MIC (µg/mL)	Vitek interpretation	Nitrocefin	Induced growth nitrocefin
1	≤0.06	S	≥0.5	R	Negative	Positive
2	≤0.06	S	0.25	R	Negative	Positive
3	≤0.06	S	≥0.5	R	Negative	Positive
4	≤0.06	S	0.25	R	Negative	Positive
5	≤0.06	S	0.25	R	Positive	Positive
6	≤0.06	S	0.12	S	Negative	Negative
7	≤0.06	S	≤0.03	S	Negative	Negative
8	≤0.06	S	≤0.03	S	Negative	Negative

^*a*^ R = Resistant; S = Susceptible.

Following discrepancy resolution and after exclusion of cephalosporin results, we observed 1 VME, 0 ME, and 14 mEs (Table 4). The VME was due to an isolate with a susceptible clindamycin MIC via Vitek 2 Compact and a resistant MIC by Sensititre COMPGP1F and did not resolve upon repeat testing. Clindamycin disk diffusion testing yielded a zone diameter of 15 mm (intermediate), and the isolate was negative by D-test for inducible clindamycin resistance, suggesting that this isolate has MICs near the clinical breakpoint and is, therefore, diagnostically challenging. The observed minocycline mE rate of 14.3% indicated validation failure and that this antimicrobial is unsuitable for routine testing and reporting in our laboratory. Of note, the Vitek 2 Compact GP81 panel recommends an alternative testing method to detect minocycline resistance, as the ability of the AST card to detect resistance was unable to be verified because resistant strains were not available at the time of comparative testing (16). The overall observations after discrepancy resolution were 97.8% EA and 98.1% CA (Tables 3 and 4).

### Nitrocefin-based detection of β-lactamase production

Nitrocefin disk testing according to manufacturer IFU using uninduced colonies resulted in 4 of the 5 penicillin-susceptible *S. pseudintermedius* isolates testing negative for β-lactamase production. However, induced growth (e.g., using growth from around a penicillin disk) nitrocefin tests confirmed all five resistant results produced by Vitek 2 Compact, resolving all MEs (Table 5).

### *S. aureus* and *S. lugdunensis*

Thirty isolates in this group were tested, including 28 *S*. *aureus* and 2 *S*. *lugdunensis* isolates (Table 6), with 0 VME, 3 ME, and 14 mE upon initial testing. One *S*. *aureus* ME occurred for clindamycin, where an isolate tested resistant by Vitek 2 Compact and susceptible by Sensititre COMPGP1F, but resolved when repeated in duplicate. Another *S. aureus* ME was due to a penicillin discrepancy where Vitek 2 Compact tested resistant and Sensititre tested susceptible; this error was resolved by performing the induced and uninduced nitrocefin tests, resulting in a susceptible interpretation. The remaining ME was a *S. lugdunensis* testing resistant to penicillin by Vitek 2 Compact and susceptible by Sensititre COMPGP1F. Nitrocefin testing using both induced and uninduced growth was performed, and the strain was negative for β-lactamase production. Three mEs (10%) were observed for minocycline, passing validation acceptance criteria. While not included in final performance calculations, 9 mEs were observed when the canine cefalothin and cefpodoxime breakpoints were used, with cefpodoxime reaching unacceptable mE rates of 26%. Again, 100% CA of oxacillin suggests reliability of oxacillin as a representative for these antimicrobials. After repeat testing, the overall observations are 99.6% EA and 98.6% CA with 0 VMEs, 1 ME, and 5 mEs (Table 3).

**TABLE 6:** *Staphylococcus aureus/lugdunensis* antimicrobial performance^*a*^^,^^*b*^^,^^*e*^

Antimicrobial	No. of isolates							Number (%)			
	Total	S	I	SDD	R	NI	EA	CA	VME	ME	mE
Penicillin^*c*^^-^Initial	30	10	0	0	2	0	96.7	93.3	0 (0)	2 (20)	0 (0)
Penicillin^*c*^-Resolved	30	10	0	0	20	0	96.7	100	0 (0)	1(3)	0 (0)
Oxacillin^*c*^	30	28	0	0	2	0	96.7	100	0 (0)	0 (0)	0 (0)
Cefalothin	30	29	1	0	0	0	100	96.7	0 (0)	0 (0)	1 (3)
Cefpodoxime	30	23	5	0	2	0	100	73.3	0 (0)	0 (0)	8 (26)
Amikacin	30	0	0	0	0	30	100	NA	NA	NA	NA
Gentamicin^*d*^	30	30	0	0	0	0	96.7	96.7	0 (0)	0 (0)	1(3)
Enrofloxacin-Canine	30	0	0	0	0	30	100	NA	NA	NA	NA
Enrofloxacin-Feline	30	30	0	0	0	0	100	100	0 (0)	0 (0)	1 (3)
Marbofloxacin-Canine	30	0	0	0	1	29	100	100	0 (0)	0 (0)	0 (0)
Marbofloxacin-Feline	30	10	0	0	0	0	100	100	0 (0)	0 (0)	0 (0)
Pradofloxacin-Canine	30	10	0	0	0	0	100	NA	0 (0)	0 (0)	0 (0)
Pradofloxacin-Feline	30	10	0	0	0	0	100	100	0 (0)	0 (0)	0 (0)
Erythromycin^*d*^	30	26	0	0	4	0	100	100	0 (0)	0 (0)	0 (0)
Clindamycin-initial	30	30	0	0	0	0	100	100	0 (0)	1 (30)	0 (0)
Clindamycin-resolved	30	30	0	0	0	0	100	100	0 (0)	0 (0)	0 (0)
Doxycycline	30	0	0	0	0	30	100	NA	NA	NA	NA
Nitrofurantoin^*d*^	30	30	0	0	0	0	100	100	0 (0)	0 (0)	0 (0)
Chloramphenicol	29	0	0	0	0	29	100	NA	NA	NA	NA
Trimethoprim/Sulfamethoxazole^*d*^	30	30	0	0	0	0	100	100	0 (0)	0 (0)	0 (0)
Minocycline	30	29	0	0	1	0	100	90	0 (0)	0 (0)	3 (10)

^*a*^ Penicillin result was resolved with induced and uninduced nitrocefin testing; clindamycin results were resolved by testing in duplicate.

^*b*^ CA = Categorical agreement; EA = Essential agreement; I = Intermediate; ME = Major error; mE = Minor error; MIC = Minimal inhibitory concentration; NA = Not applicable; S = Susceptible; SDD = Susceptible, dose dependent; R = Resistant; VME = Very major error. Results based on canine breakpoints unless otherwise specified.

^*c*^ Surrogate agent.

^*d*^ Human breakpoints.

^*e*^ Boldface values indicate errors above acceptance limits.

### *Enterococcus* spp

Thirty-four *Enterococcus* spp. isolates were tested, including 23 *Enterococcus faecalis,* 8 *Enterococcus faecium,* and 3 *Enterococcus canintestini* (Table 7). Three isolates terminated in duplicate on the Vitek 2 Compact for doxycycline and were excluded from analysis for this antimicrobial. EA for penicillin was < 90%; however, categorical agreement for penicillin was 100%, suggesting we could accept categorical results for this antimicrobial. There were no VMEs or MEs. The overall *Enterococcus* spp. observations were 96% EA, 94.9% CA, 0 VME, 0 MEs, and 6 mEs (Table 3).

**TABLE 7:** *Enterococcus* sp. antimicrobial data^*a*^^,^^*c*^

Antimicrobial	Total	No. of isolates					Number (%)				
		S	I	SDD	R	NI	EA	CA	VME	ME	mE
Penicillin^*b*^	34	32	0	0	2	0	**82.4**	100	0 (0)	0 (0)	0 (0)
Chloramphenicol	33	0	0	0	0	33	100	NA	0 (0)	0 (0)	0 (0)
Doxycycline^*b*^	31	20	0	0	0	11	100	100	0 (0)	0 (0)	0 (0)
Erythromycin^*b*^	32	8	21	0	3	0	100	90.6	0 (0)	0 (0)	3 (9)
Nitrofurantoin^*b*^	32	27	4	0	1	0	100	90.3	0 (0)	0 (0)	3 (9.6)

^*a*^ CA = Categorical agreement; EA = Essential agreement; I = Intermediate; ME = Major error; mE = Minor error; MIC = Minimal inhibitory concentration; NA = Not applicable; S = Susceptible; SDD = Susceptible, dose dependent; R = Resistant; VME = Very major error.

^*b*^ Human breakpoints.

^*c*^ Boldface values indicate errors above acceptance limits.

## DISCUSSION

 We assessed whether AST results for staphylococci and enterococci obtained by the Vitek 2 Compact were comparable to our legacy method, Sensititre BMD, to determine whether acceptable performance would be achieved for use in patient care and ultimately, veterinary AMR surveillance programs. Overall performance between the two methods was acceptable, with 96% EA and 94% CA across organism groups, yet individual performance for clindamycin, minocycline, and β-lactams in certain groups did not meet performance goals, indicating that these agents are not suitable for patient-care decisions without additional confirmatory testing. Based on acceptable performance, we believe that Vitek 2 Compact data is of sufficient quality for use in patient care and for data inclusion in veterinary AMR surveillance programs, particularly if Vitek 2 Compact AST-GP81 cards are updated to include current clinical breakpoint ranges and antimicrobials of public health importance, such as vancomycin.

 Although the overall EA and CA of the evaluation exceeded the minimum performance goal of 90%, there were individual antimicrobials that did not meet the minimum error rate specifications. A single clindamycin VME occurred for a *S. pseudintermedius* isolate and could not be resolved with repeat or ancillary testing. While it did not resolve the error in our study, offline clindamycin disk diffusion or inducible clindamycin resistance tests (18) may be an appropriate check for veterinary laboratories using Vitek-2 cards for testing. Clindamycin testing for *S. aureus/lugdunensis* failed initial analysis, but the single error resolved when testing was repeated. Minocycline testing also failed validation for *Staphylococcus* spp. not *S. aureus*/*lugdunensis* group; as such, we would advise laboratories not to report tests that fail validation. Penicillin had an unacceptable EA for *Enterococcus* spp.; however, the CA was 100%. While this could be due to inherent testing variability, methodological differences, or reader interpretation, the clinical significance is minimal as the interpretations reported to providers remain accurate. Consequently, our laboratory has decided to continue reporting penicillin on *Enterococcus* spp. from the Vitek 2 Compact.

In 2012, the CLSI Subcommittee on AST removed cephalosporin breakpoints (19) for staphylococci from CLSI M100, leaving only recommendations to routinely test either oxacillin or cefoxitin as surrogate agents for determining β-lactam susceptibility in staphylococci. The European Committee on Antimicrobial Susceptibility Testing (EUCAST) (20) also currently recommends this approach. In contrast, CLSI VET01S (11) includes breakpoints for amoxicillin-clavulanate and cephalosporins for some staphylococci. While not included in final performance calculations in our study, cefpodoxime and cefovecin for *S*. *pseudintermedius* and cefpodoxime for *S. aureus/lugdunensis* did not achieve >90% CA and also demonstrated mEs exceeding 10% when tested individually. The poor correlation of cefpodoxime between methods is consistent with 88.9% CA reported in the Vitek-2 AST-GP81 card IFU (16). Interestingly, the performance of cefpodoxime is not listed in Sensititre veterinary performance documents (21). Poor correlation of individual veterinary β-lactam clinical breakpoints to oxacillin is not unique to this study, with another Vitek-2 study using AST-GP80 cards describing individual amoxicillin-clavulanate and cefalothin testing susceptible while oxacillin tested resistant (22). In contrast, oxacillin surrogate agent inference rules resulted in 100% CA for all species of *Staphylococcus* evaluated in this study and manufacturer-reported CA of oxacillin at 99.4% and 97.1% for Sensititre panels (21) and Vitek-2 AST-GP81 cards (16), respectively. Updated panels would ideally include both oxacillin and cefoxitin, as there are bacterial species-specific recommendations (11, 17, 19, 23, 24) regarding the appropriate surrogate agent to test. We suspect that inclusion of these individual breakpoints for staphylococci in VET01S may discourage use of surrogate agent testing, add confusion surrounding result reporting for veterinary laboratories, and may confound surveillance efforts. Surrogate agents not only allow more efficient method verification but also ensure accurate patient results that can be translated across the One Health spectrum. As such, veterinary laboratories utilizing the Vitek 2 Compact AST-GP81 test panel should routinely employ oxacillin as a surrogate rather than test and report individual MICs for amoxicillin-clavulanate and cephalosporins.

Per CLSI guidance, susceptible penicillin results (≤0.12 µg/mL) in *Staphylococcus* spp. require a penicillin zone-edge or induced nitrocefin-based test for confirmation. While Vitek may have a tendency to overcall penicillin resistance, it performed more closely with confirmatory testing compared to Sensititre penicillin results. We used induced and uninduced nitrocefin tests to evaluate β-lactamase production in isolates testing penicillin susceptible by one or both methods, which resolved most of the major errors for penicillin that occurred between the Sensititre and Vitek. In line with recommendations from a previous study (23), our study emphasizes the importance of using an induced growth method (17) for accurate detection of β-lactamase production for *S. pseudintermedius*. Laboratories must use an induced nitrocefin method for confirmation of susceptible penicillin results, in accordance with CLSI VET01S and M100 guidelines, particularly if using the Sensititre COMPGP1F panel due to significant potential for reporting falsely susceptible results which might lead to treatment failure. Additionally, if used for surveillance programs, ancillary β-lactamase test results should also be collected and reported with data.

Despite being a routine AST method evaluation study, our results uniquely highlight the utilization of human guidelines in a veterinary laboratory, obsolete device formulations, challenges with our current clinical breakpoints, and resource limitations. We used the Clinical Microbiology Procedures Handbook (7) procedure for evaluation and verification of AST systems, one of two commonly used resources in human laboratories. These procedures (7, 8) are written to cover the entire evaluation process, from literature review to final determination of result acceptability. Furthermore, they are written to allow laboratories to adapt the evaluations to their patient populations and workload, making the process more accessible to smaller laboratories, including veterinary laboratories. Importantly, these procedures are also written in a format that is very familiar to technical staff and are easy to use in practice. For these reasons, we believe these procedures can be employed in veterinary laboratories for the evaluation of AST devices without undue burden on resources. By using standardized method verification protocols across sectors, we improve translatability of results leading to higher quality surveillance efforts and evaluation of resistance rates among the species.

Substantial changes to CLSI canine *Staphylococcus* spp. fluoroquinolone (enrofloxacin, marbofloxacin) and *Enterococcus* spp. and *Staphylococcus* spp. chloramphenicol breakpoints occurred during our study with publication of VET01S 7th edition (11), rendering it impossible to call the full range of results for these antimicrobials until device updates are made. Relating to surveillance, *Staphylococcus* and *Enterococcus* canine chloramphenicol clinical breakpoints (≤2, 4, ≥8 µg/mL; susceptible, intermediate, and resistant [11], respectively) may confound surveillance data translatability if the devices are updated to only include veterinary breakpoints because the updated breakpoint interpretations will call nearly all isolates resistant to chloramphenicol. Prior to canine-specific breakpoints, human chloramphenicol breakpoints (≤8, 16, ≥32 µg/mL; susceptible, intermediate, resistant) were applied to these isolates from canine patients. These changes may cause veterinary isolates to appear falsely more resistant to chloramphenicol and most resistant results will not correlate with acquired resistance mechanisms. Because these breakpoints are set in the wild-type distribution for these genera, we suspect that natural MIC fluctuation (8, 25) of ± 1 dilution and subsequent categorical disagreement during routine AST performance will result in high error rates during method evaluation procedures, meaning this antimicrobial may not be reliably tested or reported for patient care. When device updates are made, it will be important to include human ranges to ensure we can translate these data across sectors.

Currently available veterinary Sensititre COMPGP1F and Vitek 2 Compact AST-GP81 test panels do not have equivalent calling ranges for all antimicrobials; therefore, results for some antimicrobials could only be assumed to be the same and CA would be the only judge of result validity. For example, amoxicillin-clavulanate calling ranges are Sensititre (0.25–8 µg/mL) and Vitek 2 Compact (2–32 µg/mL), with Vitek 2 Compact ranges not extending to low enough concentrations to call *Staphylococcus* spp. isolates susceptible (≤0.12 µg/mL) or intermediate (0.5/0.25 µg/mL), only resistant (≥1/0.5 µg/mL) by VET01S clinical breakpoints for staphylococci (11). However, in the case of amoxicillin-clavulanate for *Staphylococcus* spp., oxacillin surrogate agent inference rules are reliable, negating the need to test amoxicillin-clavulanate for this genus.

We faced several challenges during this method evaluation. While it is appropriate to use an existing test as the reference method when verifying performance of a new test device, it must be emphasized that dried BMD panels are not equivalent to reference BMD panels. Additionally, after an in-house study, we performed QC procedures off-label for the Sensititre panel by performing QC in the same manner as patient testing to comply with good laboratory practices guidelines. Resource limitations impacted our ability to robustly evaluate aspects of device performance. We did not have the means to purchase custom frozen reference panels for error resolution. Our patient populations do not typically have unusual or high rates of resistance, and consequently, our methods comparison would have benefited from a wider variety of resistant isolates. Furthermore, the currently available AMR reference panels, such as those available from the Centers for Disease Control and Prevention ARIsolateBank for evaluation of test devices, do not include reference MICs for veterinary antimicrobials. AMR reference isolate panels including both veterinary pathogens (e.g., *S. pseudintermedius*) and veterinary antimicrobials would be an invaluable resource for laboratories.

Considering the importance of AST results and AMR detection, it is critical that AST methods used in veterinary laboratories are carefully evaluated to ensure optimal method performance so that results can be used to guide treatment, detect critical resistance mechanisms, and be translated across the One Health spectrum for more accurate determinations of AMR in bacterial pathogens. We suggest the following improvements for the benefit of all sectors: (1) Veterinary laboratory adoption of standardized AST method evaluation procedures to evaluate devices before using them for patient care or surveillance data generation; (2) Manufacturer support and investment in timely and public-health centric updates to test devices; (3) Increased vendor engagement in veterinary diagnostic tool development; (4) Production of veterinary AMR reference isolate panels for evaluation of veterinary AST devices; and (5) Adoption of alternative commercial AST method results for inclusion in veterinary AMR surveillance programs providing that the laboratory has validated the test method and appropriate antimicrobial dilution ranges are available for antimicrobials of interest. Harmonized strategies and dedication to quality data can propel veterinary laboratories toward a true One Health model of AMR detection and surveillance, leading to a greater impact for all species.
